# Association of probiotic use with nivolumab efficacy in gastric cancer patients on gastric acid suppressants

**DOI:** 10.1016/j.isci.2026.117014

**Published:** 2026-08-04

**Authors:** Mikumi Yamaguchi, Ryuji Uozumi, Tomoaki Eto, Ayumi Fujimoto, Hirotoshi Iihara, Tomoaki Hatakeyama, Ayako Higuchi, Yu Maehara, Hironori Fujii, Shinya Tamaki, Hitoshi Kawazoe, Hisakazu Ohtani

**Affiliations:** 1 Division of Clinical Pharmacy, Keio University Faculty of Pharmacy, Tokyo, Japan; 2 Department of Industrial Engineering and Economics, Institute of Science Tokyo, Tokyo, Japan; 3 Department of Pharmacy, NHO Kokura Medical Center, Fukuoka, Japan; 4 Department of Pharmacy, Osaka City General Hospital, Osaka, Japan; 5 Department of Pharmacy, Gifu University Hospital, Gifu, Japan; 6 Department of Pharmacy, KKR Sapporo Medical Center, Sapporo, Japan; 7 Department of Pharmacy, NHO Kyushu Cancer Center, Fukuoka, Japan; 8 Department of Pharmacy, Keio University Hospital, Tokyo, Japan; 9 Department of Clinical Pharmacy, Keio University School of Medicine, Tokyo, Japan

**Keywords:** gastric cancer, nivolumab, gastric acid suppressants, proton pump inhibitor, probiotics, dysbiosis, progression-free survival, overall survival

## Abstract

This study aimed to evaluate the association between concomitant probiotic use and the effectiveness of nivolumab in patients with advanced gastric cancer (GC) receiving gastric acid suppressants (GASs). In this multicenter, retrospective, observational study, conducted in Japan, medical records of consecutive patients with advanced GC treated with nivolumab between September 2017 and March 2024 were reviewed. Progression-free survival (PFS) and overall survival (OS) were estimated using the Kaplan-Meier method. Hazard ratios (HRs) and 95% confidence intervals (CIs) were estimated using the Cox proportional hazards model. Overall, 249 of the total 416 patients surveyed received GASs. After 1:2 matching, 162 patients were included in this study. The probiotic group showed significantly longer PFS and OS compared to the non-probiotic group (HR, 0.61; 95% CI, 0.43–0.87 and HR, 0.69; 95% CI, 0.48–0.99). These hypothesis-generating findings suggest that probiotics may improve the effectiveness of nivolumab in patients with GC taking GASs.

## Highlights

• Gastric acid suppressants (GASs) lower efficacy of immune checkpoint inhibitors
• Probiotics may be associated with nivolumab efficacy in advanced gastric cancer (GC)
• Benefits may be seen in patients with advanced GC receiving nivolumab and GASs
• Our results highlight the role of microbiome modulation in cancer treatment

## Graphical abstract

**Figure:**
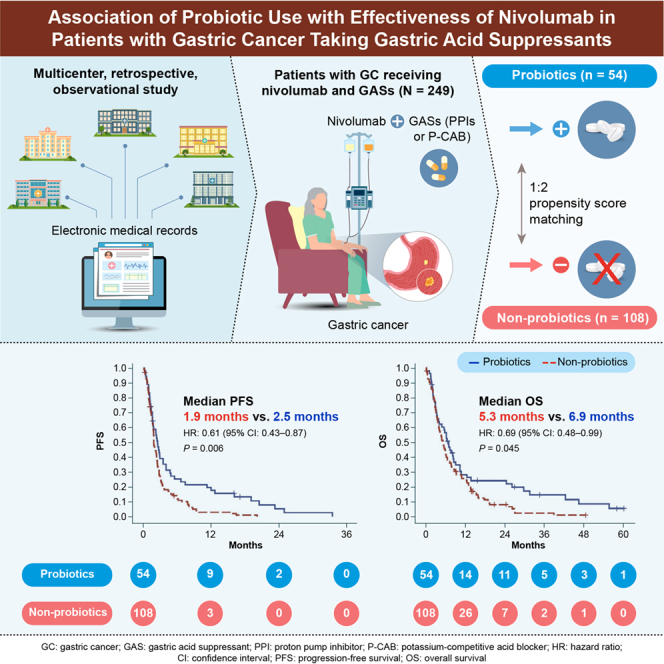

## Teaser

Health sciences; Medicine; Oncology

## Introduction

Gastric cancer (GC) is one of the most commonly diagnosed cancers in both men and women, particularly in East Asian individuals. In 2020, approximately 110,000 new cases of GC were diagnosed in Japan.^1^ Immune checkpoint inhibitors (ICIs), such as nivolumab and pembrolizumab, block the binding of programmed cell death ligand 1 (PD-L1)/ligand 2 to the programmed cell death-1 (PD-1) receptor, thereby releasing the brake on T cells and restoring their inherent antitumor activity. ICI monotherapy and the combination of ICIs and cytotoxic chemotherapy have become the standard treatment for many types of cancer, including GC.^2^^,^^3^^,^^4^ Recently, gut microbiota has attracted attention as a new therapeutic target in cancer. Emerging evidence has indicated that both the diversity and compositional structure of the gut microbiota are associated with clinical responses to ICIs.^5^^,^^6^ Fecal microbiota transplantation from ICI responders has been shown to overcome resistance to anti-PD-1 therapy in patients with melanoma.^7^^,^^8^ These findings support the concept that the modulation of the gut microbiota affects the treatment outcome of patients treated with ICIs. Gastric acid suppressants (GASs), including proton pump inhibitors (PPIs) and potassium-competitive acid blockers (P-CABs), as well as antibiotics (ATBs), are known to disrupt the gut microbiota and induce dysbiosis.^9^^,^^10^^,^^11^ They have also been associated with reduced effectiveness of ICI treatment in patients with cancer.^12^^,^^13^^,^^14^ However, no treatment strategy has been established yet for addressing this problem.

In Japan, probiotics are widely used to improve symptoms of dysbiosis, such as diarrhea, constipation, and gastrointestinal symptoms.^15^ Probiotics can improve the outcomes of patients with advanced non-small cell lung cancer (NSCLC) treated with ICIs.^16^^,^^17^^,^^18^^,^^19^^,^^20^^,^^21^^,^^22^ Some probiotics contain *Clostridium butyricum*, which is a butyrate-producing bacterium that forms spores and modulates cancer immunity. *In vivo* studies have shown that butyrate-producing bacteria can regulate the differentiation of regulatory T cells (Tregs) and enhance CD8^+^ T cell activity through the production of short-chain fatty acids (SCFAs).^23^^,^^24^^,^^25^ It has been suggested that *Clostridium butyricum* (CBM588) can restore the decreased effectiveness of ICIs in patients with NSCLC receiving PPIs and ATBs.^17^^,^^18^ Two randomized phase I trials showed prolonged progression-free survival (PFS) in patients with metastatic renal cell carcinoma (mRCC) treated with ICIs in combination with CBM588.^26^^,^^27^ However, NSCLC and mRCC are epithelial tumors, whereas GC is an adenocarcinoma, which is a different histological subtype. While the current evidence supports the potential benefits of probiotics in patients with NSCLC and mRCC, it remains unclear whether probiotics have similar effects in patients with GC treated with ICIs, with or without chemotherapy. GC is particularly common in East Asia, where the proportion of patients taking GASs is also high. However, there is a lack of evidence regarding the effect of probiotics in patients with GC in clinical settings.

Therefore, this study aimed to evaluate the association between concomitant probiotic use and the effectiveness of nivolumab in Japanese patients with advanced GC who are taking GASs.

## Results

### Patient characteristics

**Figure 1:**
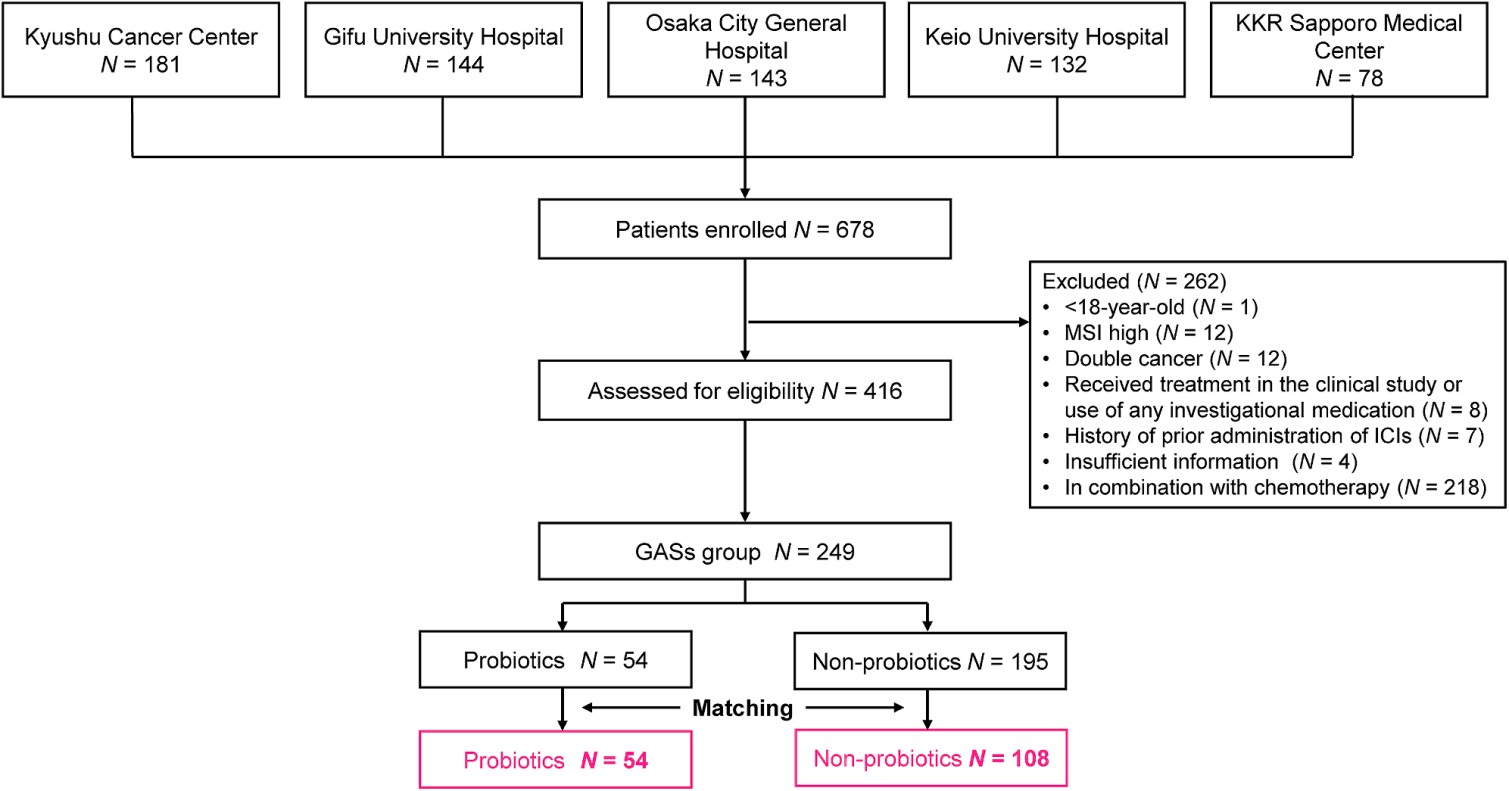
Patient enrollment flowchart PPI, proton pump inhibitor; MSI, microsatellite instability; ICI, immune checkpoint inhibitor.

Figure 1 shows the patient enrollment flowchart. Overall, 416 patients were initially enrolled. Among them, 249 patients were taking GASs. After propensity score matching, these patients were allocated to the groups treated with (probiotic group; *n* = 54) and without (non-probiotic group; *n* = 108) concomitant probiotics. Four variables included in the propensity score analysis showed an absolute standardized mean difference (SMD) between 0.02 and 0.03.

Table 1 presents the baseline characteristics of the patients. The median age of the patients was 69 years (range: 24–94 years). The proportion of male patients in the probiotic and non-probiotic groups was 64.8% and 62.0%, respectively, with an SMD of 0.06, indicating that the two groups were well-balanced in terms of sex. Approximately 80% of the patients were receiving third-line treatment. The prescribed GASs included PPIs and P-CABs, specifically lansoprazole (38.9%) in the probiotic group and vonoprazan (38.0%) in the non-probiotic group. Additionally, the prescribed probiotics were *Clostridium butyricum* (68.5%), Miya-BM (Miyarisan Pharmaceutical Co. Ltd., Japan), Bio-three (Toa Pharmaceutical Industry Co. Ltd., Japan), *Bifidobacterium* (31.5%), LAC-B (Kowa Company, Ltd., Japan), Biofermin, and Biofermin R (Taisho Pharmaceutical Co. Ltd., Japan).

**Table 1:** Patient characteristics of the original cohort and propensity score-matched cohort

Characteristics	Original cohort					Propensity score-matched cohort				
	Probiotics (*N* = 54)		Non-probiotics (*N* = 195)		SMD	Probiotics (*N* = 54)		Non-probiotics (*N* = 108)		SMD
Age (years), median (range)	69.5	(43–86)	69.0	(24–94)	0.25	69.5	(43–86)	69.0	(24–84)	0.31
Sex										
Male	35	(64.8)	122	(62.6)	0.05	35	(64.8)	67	(62.0)	0.06
Female	19	(35.2)	73	(37.4)	−0.05	19	(35.2)	41	(38.0)	−0.06
ECOG PS										
0–1	43	(79.6)	137	(70.3)	0.22	43	(79.6)	85	(78.7)	0.02
≥2	11	(20.4)	58	(29.7)	−0.22	11	(20.4)	23	(21.3)	−0.02
Treatment line										
≤3rd	42	(77.8)	159	(81.5)	−0.09	42	(77.8)	83	(76.9)	0.02
≥4th	12	(22.2)	36	(18.5)	0.09	12	(22.2)	25	(23.1)	−0.02
Previous gastrectomy										
No gastrectomy	35	(64.8)	139	(71.3)	−0.14	35	(64.8)	71	(65.7)	−0.02
Partial/total gastrectomy	19	(35.2)	56	(28.7)	0.14	19	(35.2)	37	(34.3)	0.02
HER2										
Negative	46	(85.2)	157	(80.5)	0.12	46	(85.2)	93	(86.1)	−0.03
Positive	8	(14.8)	38	(19.5)	−0.12	8	(14.8)	15	(13.9)	0.03
GASs used										
Lansoprazole	21	(38.9)	56	(28.7)	0.22	21	(38.9)	37	(34.3)	0.10
Vonoprazan	19	(35.2)	83	(42.6)	−0.15	19	(35.2)	41	(38.0)	−0.06
Esomeprazole	11	(20.4)	39	(20.0)	0.01	11	(20.4)	20	(18.5)	0.05
Rabeprazole	2	(3.7)	14	(7.2)	−0.15	2	(3.7)	8	(7.4)	−0.16
Omeprazole	1	(1.9)	3	(1.5)	0.02	1	(1.9)	2	(1.9)	0
Probiotic used										
*Clostridium butyricum* (Miya-BM)	32	(59.3)	–	–	–	32	(59.3)	–	–	–
*Bifidobacterium* (LAC-B)	8	(14.8)	–	–	–	8	(14.8)	–	–	–
*Bifidobacterium* (Biofermin)	7	(13.0)	–	–	–	7	(13.0)	–	–	–
*Enterococcus faecium*, *Clostridium butyricum*, and *Bacillus subtilis* (Bio-three)	5	(9.3)	–	–	–	5	(9.3)	–	–	–
*Bifidobacterium* (Biofermin R)	2	(3.7)	–	–	–	2	(3.7)	–	–	–

Data are presented as the number (%) of patients or SMD unless otherwise indicated. Percentages have been rounded off and may not total 100. ECOG PS, Eastern cooperative oncology group performance status; GASs, gastric acid suppressants; HER2, human epidermal growth factor receptor type 2; SMD, standardized mean difference.

### Efficacy

**Figure 2:**
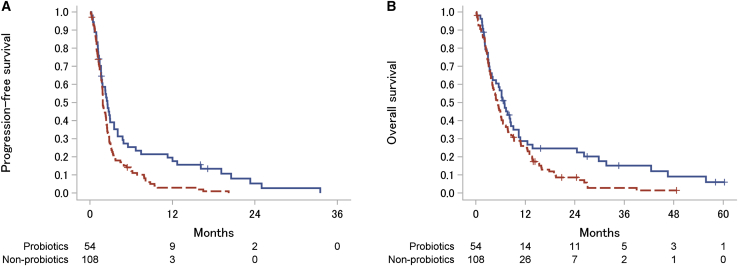
Kaplan-Meier survival curves of patients treated with nivolumab monotherapy according to whether probiotics were used concomitantly in a propensity score-matching The solid and dashed lines represent the probiotic and non-probiotic groups, respectively. The numbers at risk are shown at the bottom. (A) Progression-free survival. (B) Overall survival.

The median follow-up time was 48.7 (interquartile range, 27.0–58.1) months. The median PFS in the probiotic and non-probiotic groups was 2.5 (95% confidence interval [CI], 1.7–3.5) months and 1.9 (95% CI, 1.7–2.3) months, respectively (Figure 2A). The crude hazard ratio (HR) was 0.61 (95% CI, 0.43–0.87; *p* = 0.006) in univariable analysis. Similarly, the median overall survival (OS) in the probiotic and non-probiotic groups was 6.9 (95% CI, 3.8–8.5) months and 5.3 (95% CI, 4.0–6.2) months, respectively (Figure 2B). The crude HR was 0.69 (95% CI, 0.48–0.99; *p* = 0.045) in univariable analysis.

**Figure 3:**
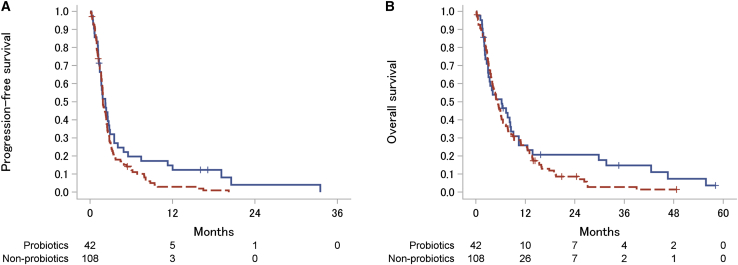
Kaplan-Meier survival curves of patients treated with nivolumab monotherapy according to whether probiotics were used before nivolumab initiation The solid and dashed lines represent the probiotic and non-probiotic groups, respectively. The numbers at risk are shown at the bottom. (A) Progression-free survival. (B) Overall survival.

**Figure 4:**
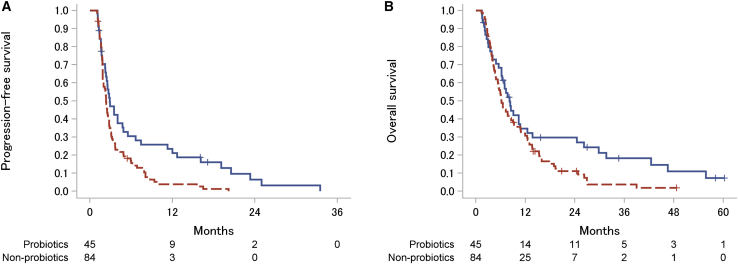
Kaplan-Meier survival curves of patients treated with nivolumab monotherapy according to whether probiotics were used concomitantly in a 30-day landmark analysis The solid and dashed lines represent the probiotic and non-probiotic groups, respectively. The numbers at risk are shown at the bottom. (A) Progression-free survival. (B) Overall survival.

In the subgroup analysis defining probiotic exposure before nivolumab initiation, probiotic use was not significantly associated with prolonged PFS or OS. Specifically, the median PFS in the probiotic (*n* = 42) and non-probiotic groups (*n* = 108) was 2.2 (95% CI, 1.6–2.8) months and 1.9 (95% CI, 1.8–2.4) months, respectively (log rank test; *p* = 0.083, Figure 3A). The median OS in the probiotic and non-probiotic groups was 6.3 (95% CI, 3.0–8.6) months and 5.4 (95% CI, 5.3–6.3) months, respectively (log rank test; *p* = 0.206, Figure 3B). Conversely, a landmark analysis showed that probiotic use was associated with prolonged PFS and OS. The median PFS in the probiotic (*n* = 45) and non-probiotic groups (*n* = 84) was 2.9 (95% CI, 2.2–4.7) months and 2.3 (95% CI, 1.9–2.8) months, respectively (log rank test; *p* = 0.006, Figure 4A). Similarly, the median OS in the probiotic and non-probiotic groups was 8.2 (95% CI, 6.2–10.8) months and 6.2 (95% CI, 5.3–8.5) months, respectively (log rank test; *p* = 0.049, Figure 4B).

**Figure 5:**
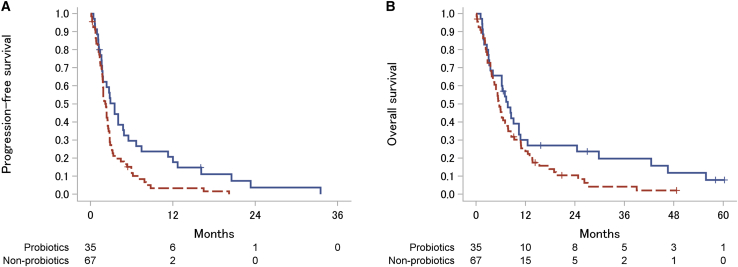
Kaplan-Meier survival curves of patients treated with nivolumab monotherapy according to whether probiotics were used concomitantly in PPI population The solid and dashed lines represent the probiotic and non-probiotic groups, respectively. The numbers at risk are shown at the bottom. (A) Progression-free survival. (B) Overall survival.

The subgroup of PPI population (*n* = 102), except for P-CAB (*n* = 60), showed similar results. The median PFS in the probiotic (*n* = 35) and non-probiotic groups (*n* = 67) was 3.5 (95% CI, 1.7–4.9) months and 2.2 (95% CI, 1.8–2.5) months, respectively (log rank test; *p* = 0.009, Figure 5A). Similarly, the median OS in the probiotic and non-probiotic groups was 7.7 (95% CI, 4.0–10.4) months and 5.6 (95% CI, 4.1–7.3) months, respectively (log rank test; *p* = 0.067, Figure 5B).

**Figure 6:**
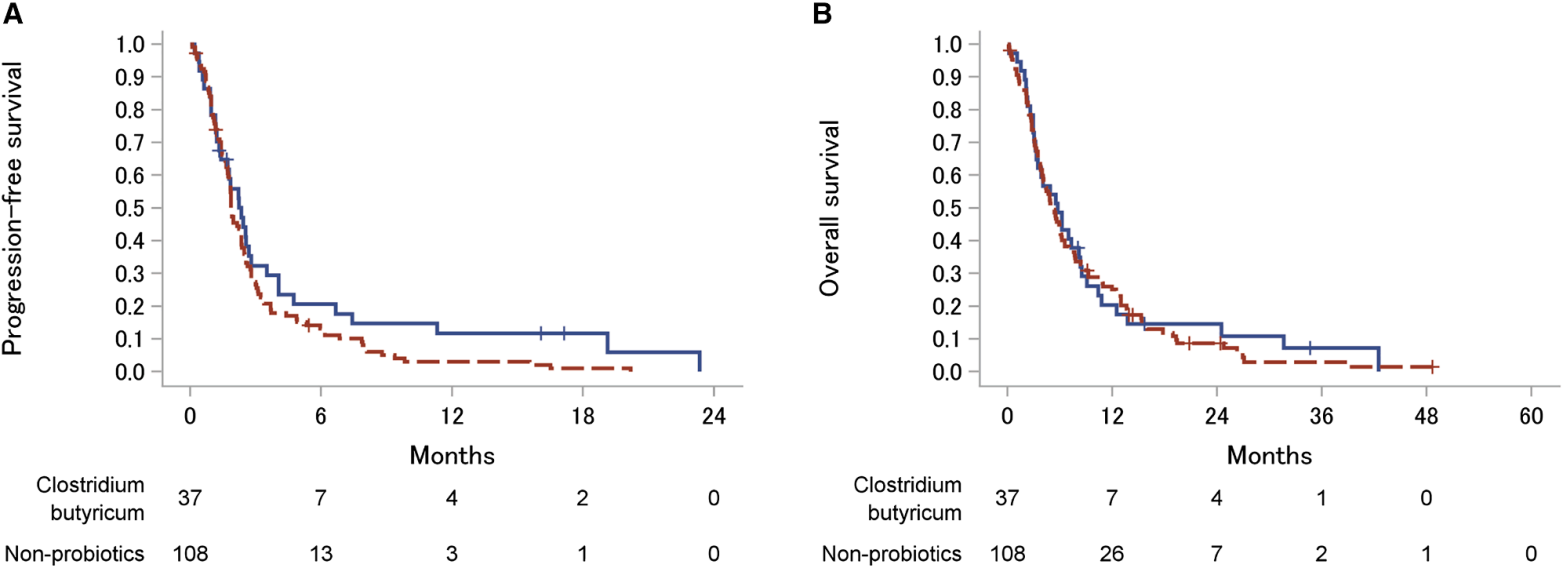
Kaplan-Meier survival curves of patients treated with nivolumab monotherapy according to whether *Clostridium butyricum* were used concomitantly The solid and dashed lines represent the *Clostridium butyricum* and non-probiotic groups, respectively. The numbers at risk are shown at the bottom. (A) Progression-free survival. (B) Overall survival.

**Figure 7:**
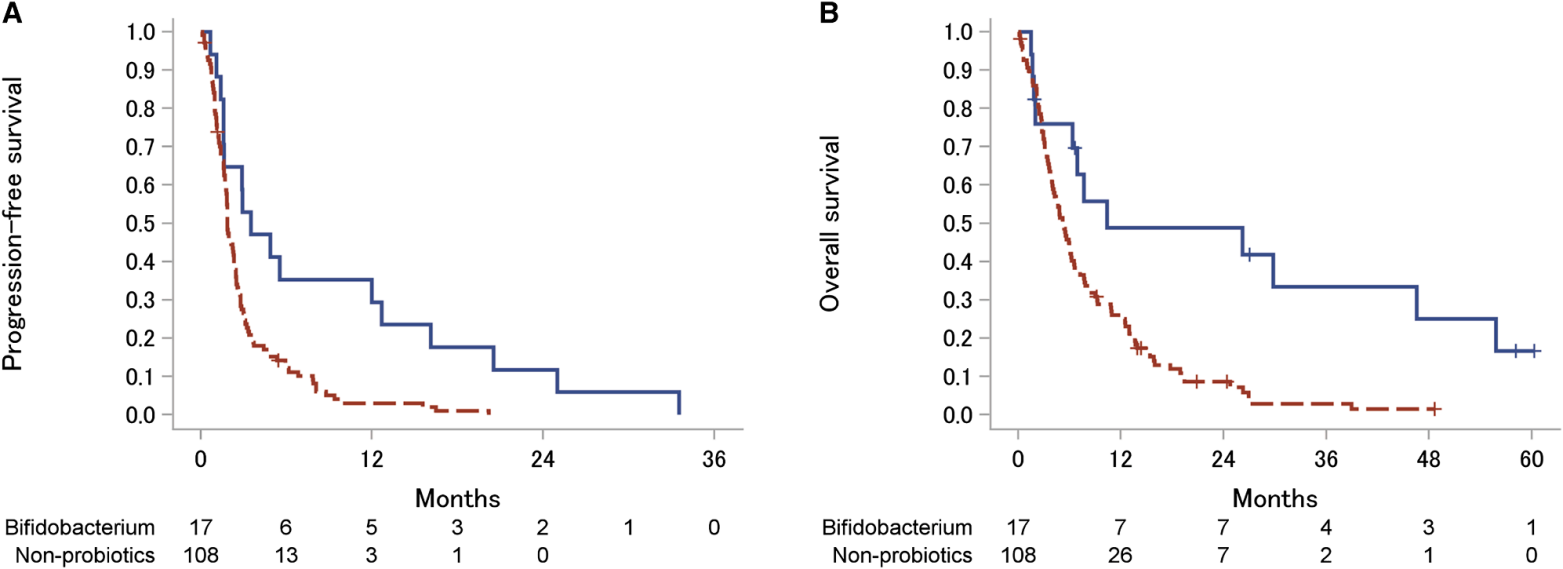
Kaplan-Meier survival curves of patients treated with nivolumab monotherapy according to whether *Bifidobacterium* were used concomitantly The solid and dashed lines represent the *Bifidobacterium* and non-probiotic groups, respectively. The numbers at risk are shown at the bottom. (A) Progression-free survival. (B) Overall survival.

Regarding the breakdown of the specific probiotic strains, *Clostridium butyricum* was not associated with the effectiveness of nivolumab. The median PFS and OS in the *Clostridium butyricum* group were 2.3 (95% CI, 1.4–2.8) months and 5.7 (95% CI, 3.3–8.3) months, respectively (Figures 6A and 6B). Conversely, *Bifidobacterium* was significantly associated with the effectiveness of nivolumab. The median PFS and OS in the *Bifidobacterium* group were 3.5 (95% CI, 1.6–12.7) months and 10.4 (95% CI, 2.0–46.6) months, respectively (Figures 7A and 7B). The crude HR of PFS and OS was 0.40 (95% CI, 0.22–0.70; *p* = 0.002) and 0.36 (95% CI, 0.18–0.66; *p* = 0.002) in univariable analysis.

As shown in Table 2, the concomitant use of probiotics showed a trend toward a reduced risk of disease progression and death between the two groups in the sensitivity analyses using a propensity score-adjusted multivariable model and inverse probability of treatment weighting (IPTW) for both PFS (adjusted HR, 0.79; 95% CI, 0.57–1.09 and adjusted HR, 0.81; 95% CI, 0.60–1.09, respectively) and OS (adjusted HR, 0.84; 95% CI, 0.60–1.18 and adjusted HR, 0.86; 95% CI, 0.62–1.19, respectively). The Bayesian posterior probability of HRs for PFS from 0.8 to 1.25 ranged from 6% to 56%. Also, the Bayesian posterior probability of HRs for OS from 0.8 to 1.25 ranged from 20% to 77%.

**Table 2:** Propensity score analysis results for PFS and OS

	Adjusted HR (95% CI)	*p* value	Posterior probability (%)^a^
PFS			
Propensity score matching	0.61 (0.43–0.87)	0.006	6
Multivariable analysis	0.79 (0.57–1.09)	0.159	47
IPTW	0.81 (0.60–1.09)	0.169	56
OS			
Propensity score matching	0.69 (0.48–0.99)	0.045	20
Multivariable analysis	0.84 (0.60–1.18)	0.315	59
IPTW	0.86 (0.62–1.19)	0.369	77

HR, hazard ratio; CI, confidence interval; PFS, progression-free survival; OS, overall survival; IPTW, inverse probability of treatment weighting.

a Computed as a Bayesian posterior probability of HRs from 0.8 to 1.25 based on non-informative prior distribution.

### Safety

The incidence rates of grade 3/4 adverse events are shown in Table 3. The most common grade 3 adverse event was liver dysfunction. No significant differences in adverse events were observed between the probiotic and non-probiotic groups (Fisher’s exact test: *p* = 0.509). No grade 5 adverse events were observed.

**Table 3:** Incidence of grade 3/4 adverse events

	Probiotics (*N* = 54)		Non-probiotics (*N* = 108)	
Any adverse event	5	(9.3)	6	(5.5)
Liver dysfunction	2	(3.7)	2	(1.9)
Rash	2	(3.7)	1	(0.9)
Diarrhea	1	(1.9)	1	(0.9)
Colitis	0	(0)	1	(0.9)
Pneumonitis	0	(0)	1	(0.9)

Data are presented as the number (%) of patients.

## Discussion

In this study, the concomitant use of probiotics was significantly associated with longer PFS and OS in Japanese patients with advanced GC treated with nivolumab monotherapy with concurrent GAS administration. Subgroup analyses indicated that the favorable effect was more apparent in patients receiving PPIs and in those administered *Bifidobacterium*-containing probiotics. Importantly, the concomitant use of probiotics did not increase the incidence of grade ≥3 adverse events, supporting the tolerability of this strategy in routine clinical practice. However, in sensitivity analyses using the propensity score-adjusted Cox model and IPTW, as well as the subgroup analysis defining probiotic exposure before ICI initiation, probiotic use was not significantly associated with prolonged PFS or OS. Our results suggest that adding probiotics after ICI initiation might be effective; further studies are needed to clarify the relationship between the timing of probiotic initiation and its effectiveness. These hypothesis-generating findings suggest that probiotics may improve the reduced effectiveness of nivolumab because of the use of GASs, at least in the context of ICI monotherapy.

The association between probiotic use and the effectiveness of nivolumab in patients with advanced GC receiving GASs is thought to be attributable to the microbiota-mediated modulation of antitumor immunity. PPI exposure reportedly increases the abundance of *Bacteroidetes*, which has an unfavorable effect on the effectiveness of nivolumab.^8^^,^^9^ In contrast, previous studies have suggested that probiotics containing *Clostridium butyricum* and SCFAs produced by this bacterium enhance CD8^+^ T cell activation.^23^^,^^24^^,^^25^ In recent years, accumulating evidence suggests that microbe-immunotherapy interactions could be categorized by the tumor immune microenvironment (TIME) mechanistic framework.^28^ *Bifidobacterium* has several roles in the TIME framework as follows: *Bifidobacterium* could enhance T cell immunity mediated by dendritic cells for immunotherapy potentiation. *Bifidobacterium* could mediate innate immunity. Natural killer cells and proinflammatory M1 macrophages play a pivotal role in innate immunity against tumors. *Bifidobacterium* could secrete the metabolite inosine. Inosine activates T helper 1 cells. Epithelial injury and immunogenic bacteria stimulate dendritic cells for tumor immunity. Consequently, the concomitant use of probiotics may be associated with antitumor immune responses and subsequently may induce the prolonged effectiveness of nivolumab in patients with GC receiving PPIs.

The present findings are broadly consistent with previous reports in NSCLC and mRCC,^16^^,^^17^^,^^18^^,^^19^^,^^20^^,^^21^^,^^22^ in which probiotics, particularly CBM588, were associated with improved ICI efficacy. Several retrospective studies in NSCLC demonstrated that probiotics attenuated the negative impact of ATBs and PPIs on ICI outcomes,^17^^,^^18^ while two prospective phase I studies in mRCC showed prolonged PFS with CBM588 combined with nivolumab-based therapy.^26^^,^^27^ However, evidence regarding GC has been extremely limited despite the high prevalence of both GC and GAS use in East Asian populations. The current study therefore extends the existing literature by suggesting that modulation of the gut microbiota may also influence ICI responsiveness in GC. Notably, the observed magnitude of benefit in this cohort was modest compared with that reported in mRCC trials, which may be attributable to differences in tumor biology, prior treatment exposure, nutritional status, or baseline gut microbiota composition among cancer types. Additionally, unlike previous studies that primarily focused on *Clostridium butyricum*,^16^^,^^17^^,^^18^ our results suggest that *Bifidobacterium*-containing probiotics may warrant greater attention as a potentially relevant adjunctive strategy during ICI treatment in GC. Our results suggest that the impact of probiotic strains may vary according to cancer type. However, we could not perform gut microbiota analyses in this study. Further studies are needed to clarify the effects of probiotic use on the gut microbiota and immune system, as well as its association with different cancer types. In the ATTRACTION-2 trial, median PFS and OS were 1.61 months (95% CI, 1.54–2.30) and 5.26 months (95% CI, 4.60–6.37) in the nivolumab group, respectively.^3^ Compared to the phase III trial of nivolumab monotherapy, this cohort had a favorable prognosis (median PFS and OS were 2.5 months and 6.9 months in the probiotic group and 1.9 months and 5.3 months in the non-probiotic group). Currently, the combination of nivolumab and chemotherapy is the first-line treatment for advanced GC, whereas nivolumab monotherapy is the third-line treatment. Further research is required to verify these findings.

To further enrich the discussion regarding the evolving landscape of GC and systemic factors influencing treatment outcomes, we should consider several recent developments. The background of GC is changing, as metabolic dysfunction-associated fatty liver disease has been significantly associated with the incidence of *Helicobacter pylori*-negative GC.^29^ Contextualization of how systemic inflammation and the gut-liver axis contribute to the GC environment and immunotherapy should be clarified in the future. This study focused on pharmacological interventions; however, the circadian rhythm is also emerging as a critical factor in immunotherapy. Recent evidence suggests that morning administration of ICI significantly improves survival outcomes compared to afternoon administration in patients with cancer.^30^^,^^31^ In this study, we were unable to obtain data on whether the administration took place in the morning or the afternoon in diverse hospitals. Given the space of the outpatient chemotherapy unit, it is not feasible to administer treatment to all patients in the morning. However, a more holistic view of optimizing ICI therapy through both biological and temporal factors should be considered in the future.

Our study has several strengths. First, this study included patients from five hospitals in Japan. Therefore, our findings may be applicable to patient populations in similar clinical settings. Second, we used propensity score matching with adjustment of the confounding factors, allowing for a more accurate estimation of the impact of probiotics. Third, the follow-up period was relatively long in the nivolumab monotherapy group, and we evaluated both the PFS and OS. Fourth, our findings suggest that concomitant medications, particularly those administered around the initiation of ICI therapy, may have influenced the treatment outcomes since we defined treatment groups as patients who received GASs and probiotics within a window of 30 days before and 30 days after the initiation of ICI therapy. Further studies are warranted to determine the optimal dosage and timing for probiotic administration.

### Limitations of the study

This study has some limitations. First, as this was a retrospective observational study, unmeasured confounders, such as PD-L1 expression levels, claudin-18 splice variant 2, baseline nutritional status, concomitant drugs of ATBs, corticosteroids, or other microbiome-modulating drugs, metastatic sites, prior regimens, indication bias of probiotics, and information bias, cannot be excluded. The present study focused on nivolumab monotherapy as a third- or later-line in patients with advanced GC. Companion diagnostic of PD-L1 expression levels is not mandatory in practice. Additionally, nivolumab is effective even when the combined positive score is < 1. Concomitant drugs may be diverse. Our previous study showed that patients receiving concomitant ATBs were the minority compared to those receiving concomitant GASs (16.7% vs. 54.8%).^32^ Thus, we focused on PPIs, P-CAB, and probiotics, which commonly have both negative and positive impacts on immunotherapy.^14^ Although indications for probiotics could not be identified in this retrospective study, these may include diarrhea, constipation, gastrointestinal symptoms, ATB-associated symptoms, poor nutritional status, or other clinical conditions related to prognosis. Our previous study has shown that concomitant use of probiotics was significantly associated with worse OS in patients with advanced esophageal cancer treated with nivolumab or pembrolizumab, and that this phenotype might be associated with indication bias.^33^ Nevertheless, our results showed that the prescription of probiotics—which could be considered unfavorable—was correlated with prolonged survival outcomes. Second, the sample size, particularly in subgroup analyses of specific probiotic strains, was relatively small and may have limited the statistical precision of the estimates. Third, although GASs and probiotics are oral medications, we were unable to collect data on patient adherence. In Japan, community pharmacies provide thoughtful guidance to outpatients regarding oral medications. Therefore, we consider that our patients are sufficiently well-educated to take the oral medications. Fourth, we could not investigate the indications for probiotic use or the administered doses. Differences in the prescription and dosage of probiotics among institutions might have affected the results. Fifth, this study identified the phenotype in clinical practice. Detailed microbiota analyses and measurements of SCFAs were not performed; therefore, the direct relationship between microbial alterations and clinical outcomes could not be confirmed. We are planning preclinical research to clarify the relationship between gut microbiota and immune response and those mechanisms in future investigations. Therefore, future prospective studies are warranted to elucidate the mechanisms by which concomitant probiotic use may be associated with the effectiveness of ICIs and clarify causality.

In conclusion, these exploratory findings suggest that concomitant probiotic use may improve the reduced effectiveness of nivolumab in Japanese patients with advanced GC receiving GASs, although residual confounding remains a central limitation. Accordingly, the findings should be regarded as hypothesis-generating rather than confirmatory. Probiotics may represent a feasible supportive strategy to mitigate the potential negative impact of GASs on nivolumab efficacy in patients with advanced GC. Given the widespread use of PPIs and P-CABs in practice, interventions targeting the gut microbiota may become increasingly important in optimizing immunotherapy outcomes. The findings also highlight the possibility that the effects of probiotics may be strain specific, with *Bifidobacterium* potentially playing a more prominent role than *Clostridium butyricum* in GC. Future prospective randomized controlled trials incorporating longitudinal microbiota profiling, metabolomic analyses, and immune monitoring are warranted to validate these findings and clarify the mechanisms underlying probiotic-mediated enhancement of ICI efficacy.

## Resource availability

### Lead contact

Requests for further information and resources should be directed to and will be fulfilled by the lead contact, Hitoshi Kawazoe (kawazoe-ht@keio.jp).

### Materials availability

This study did not generate new, unique reagents.

### Data and code availability

• The data presented in this article cannot be shared publicly because of the privacy concerns of the individuals who participated in the study. The data will be shared by the lead contact upon reasonable request.
• This paper does not report original code.
• Any additional information required to reanalyze the data reported in this paper is available from the lead contact upon request.

## Acknowledgments

We are grateful to all patients and medical staff at Keio University Hospital, National Hospital Organization Kyushu Cancer Center, Osaka City General Hospital, Gifu University Hospital, and KKR Sapporo Medical Center for their participation in this study. We also thank Ms. Ayuko Imaoka for the valuable support. We would like to thank Editage (www.editage.com) for English language editing and their assistance in creating the graphical abstract for this paper. This work was supported, in part, by the Keio University Academic Development Fund (to H.K./2025/04), Japan. The funding sources had no role in the study design; collection, analysis, and interpretation of the data; writing of the manuscript; or the decision to submit the manuscript for publication. No writing assistance was provided.

## Author contributions

Conceptualization, H.K.; investigation, M.Y., T.E., A.F., H.I., T.H., A.H., Y.M., H.F., and S.T.; writing – original draft, M.Y. and H.K.; writing – review and editing, T.E., A.F., H.I., T.H., A.H., Y.M., H.F., S.T., and H.O.; funding acquisition, H.K.; supervision, H.O.; formal analysis, M.Y., R.U., and H.K.; design, H.K.

## Declaration of interests

R.U. received consulting fees from Eisai Co. Ltd., Sawai Pharmaceutical Co. Ltd., SBI Pharmaceuticals Co. Ltd., Daiichi Sankyo Co. Ltd., Statcom, EPS Corporation, and Toregem Biopharma Co. Ltd., as well as honoraria for lectures from Janssen Pharmaceuticals Inc., Nippon Kayaku Co. Ltd., and SAS Institute Japan Ltd. outside of the submitted work. T.E. received consulting fees from Astellas Pharma Co. Ltd. and Nippon Kayaku Co. Ltd., as well as honoraria for lectures from Astellas Pharma Inc., Nippon Kayaku Co. Ltd., Taiho Pharmaceutical Co. Ltd., Bristol Myers Squibb Co., Novartis Pharmaceuticals Corporation, Chugai Pharmaceutical Co. Ltd., MSD, Johnson & Johnson, Genmab, and Becton Dickinson outside of the submitted work. H.I. received consulting fees from Astellas Pharma Co. Ltd. and Daiichi Sankyo Co. Ltd., as well as honoraria for lectures from Astellas Pharma Co. Ltd., Chugai Pharmaceutical Co. Ltd., Daiichi Sankyo Co. Ltd., Eli Lilly and Company, Genmab A/S, Gilead Sciences, Inc., Nippon Kayaku Co. Ltd., Sawai Pharmaceutical Co. Ltd., Taiho Pharmaceutical Co. Ltd., and Takata Pharmaceutical Co. Ltd. outside of the submitted work. T.H. received honoraria for lectures from Chugai Pharmaceutical Co. Ltd. and Ono Pharmaceutical Co. Ltd. outside of the submitted work. A.H. received honoraria for lectures from Taiho Pharmaceutical Co. Ltd., Kyowa Kirin Co. Ltd., Sawai Pharmaceutical Co. Ltd., and Eisai Co. Ltd. outside of the submitted work. H.F. received honoraria for lectures from Daiichi Sankyo Co. Ltd., Kyowa Kirin Co. Ltd., Ono Pharmaceutical Co. Ltd., and Chugai Pharmaceutical Co. Ltd. outside of the submitted work. S.T. received honoraria for lectures from Daiichi Sankyo Co. Ltd., Nipro Co. Ltd., Nippon Kayaku Co. Ltd., TOAEIYO Ltd., AstraZeneca K.K., Eli Lilly and Company, Taiho Pharmaceutical Co. Ltd., Chugai Pharmaceutical Co. Ltd., Kyowa Kirin Co. Ltd., Otsuka Pharmaceutical Factory, Inc. outside of the submitted work. H.K. received honoraria for lectures from Nippon Shinyaku Co. Ltd. outside of the submitted work.

## Declaration of generative AI and AI-assisted technologies in the writing process

During the preparation of this manuscript, the authors used ChatGPT-5.5 Instant in order to improve readability and language. After using this tool, the authors reviewed and edited the content as needed and take full responsibility for the content of the publication.

## STAR★Methods

### Key resources table

**Table:** 

REAGENT or RESOURCE	SOURCE	IDENTIFIER
**Software and algorithms**		

SAS version 9.4	SAS Institute Inc.	https://www.sas.com/offices/asiapacific/japan/platform/sas9/
JMP Student Edition version 19.1.2	SAS Institute Inc.	https://www.jmp.com/ja/academic/jmp-student-edition
SPSS Statistics version 30	IBM	https://www.ibm.com/jp-ja/products/spss-statistics

### Experimental model and study participant details

This multicenter, retrospective, observational study was conducted at five hospitals in Japan: Keio University Hospital (Tokyo), National Hospital Organization Kyushu Cancer Center (Fukuoka), Osaka City General Hospital (Osaka), Gifu University Hospital (Gifu), and KKR Sapporo Medical Center (Sapporo). Patient data were collected from the electronic medical records of each institution and subsequently integrated and analyzed at the Keio University Faculty of Pharmacy (Tokyo).

The eligibility criteria were as follows: 1) male and female patients aged ≥18 years; 2) patients diagnosed with unresectable advanced GC; and 3) consecutive patients treated with nivolumab monotherapy (240 mg intravenously every 2 weeks or 480 mg every 4 weeks) or nivolumab combined with chemotherapy, for the first time between September 2017 and March 2024. The clinical follow-up was modified at the discretion of the clinician according to the treatment efficacy and/or safety profile of each patient.

The exclusion criteria were as follows: 1) refusal to allow the use of their medical records; 2) insufficient or missing information; 3) microsatellite instability-high; 4) comorbid cancers during active treatment; 5) prior administration of ICI monotherapy or a combination of ICI and chemotherapy before the investigation period; 6) received treatment in a clinical study or use of any investigational medication that could affect the treatment outcomes during ICI treatment; or 7) nivolumab combined with chemotherapy.

The study protocol was representatively approved by the Ethics Committee of Keio University School of Medicine (approval number: 20241052). Permission to conduct this study was obtained from each facility. This study was conducted in accordance with the principles of the Declaration of Helsinki and the Ethical Guidelines for Medical and Health Research involving Human Subjects by the Ministry of Education, Culture, Sports, Science, and Technology, and the Ministry of Health, Labour, and Welfare of Japan. The requirement for written informed consent was waived by the ethics review committee due to the retrospective design of this study. Accordingly, we provided patients with an opt-out option on the official website of each hospital.

### Method details

Patient data were de-identified and analyzed anonymously. The collected data included age, sex, Eastern Cooperative Oncology Group performance status, treatment line, history of gastrectomy, medical history (including prescribed PPIs, P-CAB, and probiotic use), human epidermal growth factor receptor type 2 status, date of progression and/or death at the time of initiation of ICIs, and the incidence of adverse events of grade ≥3 while receiving ICIs. Progressive disease (PD) was defined as the first incidence of disease progression based on computed tomography findings using the Response Evaluation Criteria in Solid Tumors criteria version 1.1,^34^ or clinical progression. Adverse events were graded according to the Common Terminology Criteria for Adverse Events version 5.0.^35^ The treatment group included patients who received GASs and probiotics at any time within a window of 30 days before and 30 days after the initiation of ICI therapy. This timeframe was determined based on a previous publication.^17^ The follow-up period ended on October 31, 2024.

The primary and secondary endpoints of efficacy were PFS and OS, respectively, in patients treated with or without probiotics. Regarding the difference among GASs as well as the specific probiotic strains, PPI population, *Clostridium butyricum*, and *Bifidobacterium* were subsequently compared with non-probiotics. The safety endpoint was the incidence of grade ≥3 adverse events in patients treated with or without probiotics. PFS was defined as the period from the date of first administration of ICIs to the date of PD or death from any cause. OS was defined as the period from the date of first ICI administration to the date of death from any cause. Patients without documented PD or those who were still alive were censored for PFS and OS, respectively, on the date of the last follow-up.

### Quantification and statistical analysis

The patients’ baseline characteristics were summarized using descriptive statistics. Although the potential impact of sex or gender on the study outcomes was not directly evaluated, such an effect is considered unlikely based on general clinical consensus. To minimize potential selection bias, we performed a propensity score matching analysis. The propensity score of concomitant use of probiotics was estimated using a multivariable logistic regression model adjusting for ECOG PS (0–1 vs. ≥2), treatment line (≤3 vs. ≥4), previous gastrectomy (yes vs. no), and HER2 status (negative vs. positive).^36^ Matching was performed with the use of a 1:2 matching without replacement, with a caliper width equal to 0.2 of the standard deviation of the logit of the propensity score.^37^ Baseline balance was assessed using the standardized mean difference. In the sensitivity analyses, two propensity score-adjusted analyses were also performed: (1) a multivariable Cox proportional hazards model, which included the propensity score for the concomitant use of probiotics as a covariate, and (2) the IPTW method.^38^ PFS and OS were estimated using the Kaplan–Meier method. As a complementary evaluation, a subgroup analysis defining probiotic exposure strictly before nivolumab initiation and a landmark analysis at 30 days after nivolumab initial administration were performed to address the immortal time bias due to the exposure definition of concomitant drugs and to verify the result.

The results are presented as HRs and 95% CIs. As a post hoc analysis, the Bayesian posterior probability of HRs from 0.8 to 1.25 based on non-informative prior distribution was computed to assess the equivalence.^39^ The follow-up time was calculated using the reverse Kaplan–Meier estimate.^40^ All statistical analyses were performed using SAS version 9.4 (SAS Institute Inc., Cary, NC, USA), JMP Student Edition version 19.1.2 (SAS Institute Inc., Cary, NC, USA), and SPSS Statistics version 30 (IBM, Armonk, NY, USA). Because this was a retrospective observational study, a formal sample size calculation was not performed. All *p*-values were two-sided, and the significance level was set at 0.05.
